# Developing Canadian Defined Daily Doses for Animals: A Metric to Quantify Antimicrobial Use

**DOI:** 10.3389/fvets.2019.00220

**Published:** 2019-07-17

**Authors:** Angelina L. Bosman, Daleen Loest, Carolee A. Carson, Agnes Agunos, Lucie Collineau, David F. Léger

**Affiliations:** ^1^Centre for Food-borne, Environmental and Zoonotic Infectious Diseases, Public Health Agency of Canada, Guelph, ON, Canada; ^2^Population Medicine, Ontario Veterinary College, University of Guelph, Guelph, ON, Canada; ^3^Public Health Risk Sciences Division, National Microbiology Laboratory, Public Health Agency of Canada, Guelph, ON, Canada

**Keywords:** DDDvet, antimicrobial usage, veterinary, Canada, surveillance

## Abstract

Antimicrobial use surveillance data need to be analyzed and reported in a standardized and harmonized way. In veterinary medicine, one approach is to use defined daily doses (DDD) for animals. DDD for animals are technical standards used in various measures or metrics that quantify antimicrobial use. The European Medicines Agency published principles for assigning DDDvet values based on information on dosing obtained from nine European countries. For measuring antimicrobial use in livestock within Canada, DDDs for animals reflective of Canadian veterinary antimicrobial use (DDDvetCAs) were needed. Our objectives were (1) to describe the development of DDDvetCA standards for pigs and poultry (broiler chickens and turkeys) for authorized and compounded antimicrobial active ingredients used in Canada, including those used extra-label; and (2) to compare the DDDvetCAs with EMA's DDDvets, where possible. Species-specific DDDvetCAs were assigned based on the average of unique antimicrobial daily doses obtained from product information, stratified by route of administration and age indication (where applicable). The feed, water and bolus DDDvetCAs were compared to oral DDDvets, and injectable DDDvetCAs to parenteral DDDvets, that matched by antimicrobial active ingredient. Seventy-five DDDvetCAs were assigned for pigs; 51 for poultry. Seventeen injectable DDDvetCAs could be compared to 14 EMA's parenteral DDDvets and 53 feed, water, and bolus DDDvetCAs could be compared to 40 oral DDDvets. Feed and water DDDvetCAs were generally lower than EMA's oral DDDvets, although differences in methodology between Canada and Europe make comparisons challenging. The assignment of DDDvetCAs was a resource intensive and iterative process. EMA's published principles for assigning DDDvets were an invaluable source of information. The use of DDDvetCAs will reflect exposure of Canadian animals to antimicrobials, be useful for evaluating associations between use and resistance within Canada and provide information for risk assessment and stewardship policies. However, when reporting antimicrobial use data internationally, using the same DDD standards as other reporting countries will facilitate between country comparisons, although differences in which antimicrobial active ingredients are licensed between countries may create challenges. Future steps include assigning DDDvetCAs for other food animal species, such as cattle, veal, and farmed fish.

## Introduction

Antimicrobials have an important role in food animal production. Their use to treat, control, and prevent infections plays a part in the sustainability of food animal production (1). However, antimicrobial use (AMU), in both humans and animals, has led to the emergence of antimicrobial resistance (AMR), with a subsequent increased incidence of infections that are more difficult to treat (2). These infections have significant impacts on humans, with an estimated 700,000 people globally dying every year of drug-resistant infections in the world (2), and likely a significant impact on animals, though this information is not often reported.

For these reasons, some countries conduct surveillance of antimicrobials used in animals (3–5). The Public Health Agency of Canada's Canadian Integrated Program for Antimicrobial Resistance Surveillance (CIPARS) conducts AMU surveillance in food animals (5). These surveillance activities align with various national and international initiatives and action plans to address the threat of AMR (6–8). CIPARS currently reports information provided by the Canadian Animal Health Institute on the quantities of antimicrobial agents distributed for use in animals. For 2018 data, CIPARS will be reporting on antimicrobials sold for use in animals. This data, collected under new legislative authority, will be provided by pharmaceutical manufacturers, importers, and compounders. At the farm level, CIPARS currently collects information on AMU and AMR in grower-finisher pigs, broiler chickens, and turkeys, with the aim to expand surveillance into other food animal sectors (5). This information is used to fulfill the objectives of the CIPARS farm surveillance component which are to monitor trends in antimicrobial use in select species of livestock (5).

Data gathered by AMU surveillance programs must be analyzed and reported in a standardized and harmonized way to draw conclusions that are as accurate as possible. In addition to monitoring trends in AMU, these data are needed to develop effective farm and veterinary interventions, inform antimicrobial stewardship, and to provide information for risk assessment.

One approach for animal AMU analysis and reporting is to apply a defined daily dose (DDD) for animals. The DDD for animals is a technical unit of measurement developed by the European Medicines Agency's (EMA) European Surveillance of Veterinary Antimicrobial Consumption (ESVAC) project (9). ESVAC coined the term DDDvet to describe their DDD for animals, which are used in various metrics to quantify AMU (9). The DDD are used to adjust the kilograms of active antimicrobial ingredients (AAIs) by the daily dose of the antimicrobial, measured in mg per kilogram of animal (9). This concept is based on the globally accepted DDD in human medicine (10).

The creation (or assignment) of standardized DDD for animals involves determining an average dose for each AAI authorized for use in the species of interest by route of administration (9). The principles of DDD assignment may also be extended to AAIs authorized for use in another species and used in an extra-label manner in the species of interest. As technical standards, these assigned DDDs are not meant to be considered recommended doses and may not represent doses that are used in practice (9). Instead, the assigned DDDs simply provide standard doses that can be used to facilitate standardized measurements of AMU. These standardized measurements can be used to examine trends in AMU over time, to compare of AMU between different regions, across species, and different AAIs, and to examine associations between AMU and AMR (9).

Accounting for dose when analyzing and reporting AMU is important since dosing between antimicrobials varies. This variation in dose may be due to differences in mechanism of action, pharmaceutical formulations, and metabolism and distribution in the body (11). To demonstrate the importance of accounting for dose when comparing antimicrobial use on two farms, we have provided the following hypothetical example. During one production cycle, farm A gives a single injection of ceftiofur to 100 grower-finisher pigs at 3 mg/kg, and farm B gives a single injection of tiamulin to 100 grower-finisher pigs at 11 mg/kg. Both farms have 1,000 grower-finisher pigs (with an average weight at treatment of 65 kg). Farm A used 19,500 mg of ceftiofur, which equals 0.3 mg per kg of animal, while farm B used 71,500 mg of tiamulin, equal to 1.1 mg/kg of animal. From a weight perspective, farm A appears to have used less antimicrobial than farm B, yet each farmer administered the same number of treatments to the same number of animals. If we adjust the kilograms of antimicrobial used by the dose, we find that both farm A and B used 6,500 DDD for animals (kg). Another way to interpret this value is say that both farms treated 6,500 kg of pig with one daily dose of antimicrobial. By adjusting the weight of the antimicrobial given by its DDD for animals, comparisons in use between antimicrobials with different doses are more informative.

In 2015, the EMA published principles for assigning DDDvets (9) with the goal to harmonize where possible with the methodology published by the World Health Organization (10). EMA's published principles for assigning DDDvets were invaluable in informing and guiding this project (9). These principles for assigning DDDvets were followed in 2016 by the publication of EMA assigned DDDvets for pigs, cattle and poultry, based on product information on dosing for veterinary antimicrobials obtained from Summaries of Product Characteristics (SPC) from nine European countries (12). Prior to the publication of DDDvets by the EMA, Postma et al. (13) had described assigning defined daily dose animal (DDDA) for each antimicrobial product authorized for use in pigs, using product information from four European countries. Other countries have developed national DDD for animals, including the Netherlands and Denmark, although different terminology is used to describe them (14, 15).

Using the principles for assigning DDDvets published by EMA, CIPARS decided to develop Canadian DDD for animals (DDDvetCAs). The decision to develop DDDvetCAs was made because of expected differences in antimicrobials between Canada and other countries, including antimicrobials registered for use, antimicrobial doses, the number of unique doses for an antimicrobial, and indications for use. Standardized DDD for animals based on antimicrobials authorized for use in Canada were needed for the analysis of AMU within a Canadian context. The primary objective of this study was to develop DDDvetCAs for all antimicrobials authorized (or otherwise known to be used in an extra-label manner) for use in Canada, starting with pigs and poultry (including broiler chickens and turkeys). A secondary objective was to compare DDDvetCAs with EMA's DDDvets for both species.

## Methods

We used the EMA's *Principles for the assignment of defined daily doses for animals* as guidance in the assignment of DDDvetCAs for pigs and poultry, with minor changes as required (9).

### Collection of Antimicrobial Daily Dose Information

Decisions about which antimicrobials and which doses to include in the assignment of DDDvetCAs differed in some ways to decisions made by EMA. In contrast to EMA, we assigned DDDvetCAs to coccidiostats and ionophores, as farm surveillance data about their use are collected and they are classified in Canada as antimicrobials. Also included were antimicrobials with growth promotion properties, such as bacitracin, virginiamycin, and avilamycin. Compounded antimicrobials with no equivalent authorized product in the species of interest and those used in an extra-label manner were also included, with evidence of use from Canadian surveillance data. Extra-label drug use was defined as use of an antimicrobial in a species or by route of administration not described on the label.

Daily doses from antimicrobial products authorized for use in Canada were obtained from product information found in the Canadian Compendium of Veterinary Products (16) and the Compendium of Medicating Ingredient Brochures (17). Information on doses for compounded antimicrobials were obtained from a survey in one province, that collected the prescribed dose from the label applied to the product by the veterinarian (Cécile Ferrouillet, personal communication, 2017). Doses for antimicrobials used in an extra-label manner were obtained from expert opinion (Agnes Agunos, personal communication, 2016; Anne Deckert, personal communication, 2016).

Microsoft Excel® (2010) was used to tabulate the unique daily doses for each AAI, regardless of indication. Doses were stratified by species and route of administration (in feed, in water, by injection, and by individual oral treatment or bolus). While EMA chose to group oral routes of administration together, we chose to keep them in separate categories. Since product information for feed and water medications often include doses in units such as mg/kg feed or mg/L water, conversion to mg/kg body weight was required. To do so, these doses were multiplied by either the feed or water to weight conversion ratio (in kg feed per kg animal or in L water per kg animal), as per ESVAC (9). Also, in most cases, only treatment and prevention doses were tabulated; growth promotion doses were excluded, except where the only doses available for an antimicrobial were for growth promotion purposes. This exception applied to most coccidiostats and ionophores in pigs, and to bambermycin and benzylpenicillin used in feed in poultry. These doses were clearly labeled as growth promotion doses in order to clearly identify where they were used.

**Figure 1:**
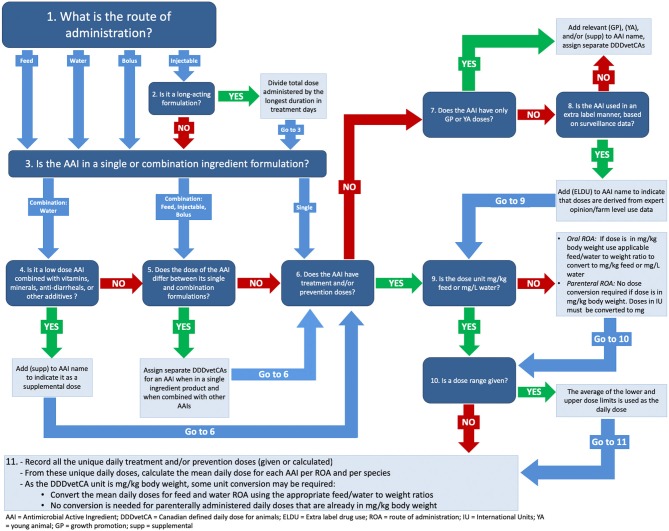
A decision tree illustrating the process of assigning Canadian defined daily doses for animals (DDDvetCAs).

Due to heterogeneity in drug product information, decisions had to be made during the tabulation of product doses (Figure 1). If the product information recommended an initial loading dose followed by a maintenance dose, the daily dose was determined by calculating the total dose given over the recommended number of days of treatment, divided by the recommended number of days. For combination products containing more than one AAI, the daily dose of each AAI in the combination was determined by multiplying the dose of the combination product by the proportion of each AAI in the product. If a dose range was reported, the mean of the range was used to assign the DDDvetCA. If the dose was expressed in international units, the dose was converted to mg with conversion factors used by the EMA (18). To obtain a daily dose for long-acting injectable products, the dose was divided by either the duration of action (in days) if available, or the recommended dosing frequency (e.g., every 3 days) if the duration of action was not available. In some instances, we contacted product manufacturers to get information about durations of action when the product information was unclear (i.e., tulathromycin, and benzathine benzylpenicillin, and procaine benzylpenicillin). When the concentration of an AAI was reported as both a salt and a base, the salt concentration was used to calculate the daily dose. For example, an in-feed product containing tiamulin for use in pigs indicated that 1 kg of product contained 100 g of tiamulin hydrogen fumarate (the salt), equaling 80.9 g of tiamulin base. In this case, the salt concentration of 100 g/kg was used to calculate the daily dose.

The distribution of doses was examined by calculating the minimum, maximum, and median daily doses for each AAI, in addition to the mean daily dose. To compare the mean daily dose (DDDvetCA) to the median dose, a ratio was calculated using Equation (1).

(1) $$\begin{array}{r} Ratio\;mean:median\;dose=DDDvetCA/\;median\;dose \end{array}$$

### Additional Information Recorded

In addition to dose information, the Health Canada Category of the AAI was recorded (19). These categories include Category I—Very high importance (to human medicine), Category II—High importance, Category III—Medium importance and Category IV—Low importance (19). These categories are distinguished by their importance in human medicine and the availability of effective alternatives should resistance occur. Health Canada category I and II antimicrobials are used to treat serious infections in humans, however, if resistance to category II antimicrobials occurs, category I antimicrobials could be substituted; there are no substitutes for antimicrobials in category I (19). Category III antimicrobials are less essential due to the availability of alternatives in categories I and II (19). Category IV antimicrobials include flavophospholipols and ionophores, which are not currently used in human medicine (19).

### Assignment of DDDvetCAs

The DDDvetCAs, in mg/kg animal body weight per day, were assigned by calculating the mean of the tabulated unique daily doses for each AAI, stratified by species and route of administration. Examples of mean daily dose calculations can be found in Table 1. The mean daily dose was assigned as the DDDvetCA for that antimicrobial and route of administration. Generally, an AAI was assigned one DDDvet per route of administration, except for some AAIs used in combination. Following ESVAC's guidelines, when the dose of an AAI differed between single and combination ingredient use, due to a synergistic effect, the AAI was assigned two separate DDDvetCAs (9). One DDDvetCA was assigned based on the daily dose for single ingredient use and a second DDDvetCA was assigned based on the daily dose for combination ingredient use. For example, in poultry, the single ingredient formulation dose for lincomycin in water was 16 mg/L, while the lincomycin-spectinomycin formulation dose for lincomycin in water was 277.5 mg/L (16). In this case, two DDDvets were calculated, one for single use lincomycin, and one for lincomycin when administered as a combination product containing both lincomycin and spectinomycin. We followed ESVAC's convention of identifying DDDvets for antimicrobials used in combination as: *antimicrobial 1_antimicrobial 2*, which is understood as the DDDvetCA for antimicrobial 1 when it is used in combination with antimicrobial 2. In a similar situation, long-acting injectable ceftiofur in pigs was assigned a separate DDDvetCA from conventional injectable ceftiofur as the daily dose between the two formulations differed (1 and 3 mg/kg, respectively). Finally, consistent with EMA guidelines (9), we assigned prodrugs their own DDDvetCAs (e.g., procaine benzylpenicillin).

**Table 1:** Example calculation of the mean daily dose and the Canadian defined daily dose for animals (DDDvetCA) for selected antimicrobial active ingredients and routes of administration, in poultry and pigs.

**Species**	**Antimicrobial active ingredient**	**ROA ^a^**	**Number of products marketed in Canada^b^**	**Unique dosages**	**Mean daily dose**	**Feed or water WCR^c^^,^^d^**	**DDDvetCA (mg/kg BW/day)^e^**
Pigs	Chlortetracycline	Feed	12	55, 110, 220, 656	260.3 mg/kg feed	0.04	10.4
	Tylosin	Injectable	1	5.5	5.5 mg/kg bodyweight	N/A^f^	5.5
	Tylosin	Water	2	83, 250	166.5 mg/L water	0.1	16.7
Poultry	Bacitracin	Feed	3	55, 82.5, 110	77.9 mg/kg feed	0.13	10.1
	Amoxicillin	Water	2	52	52.0 mg/L water	0.23	12.0

a Route of administration.

b Canadian Animal Health Institute (16).

c WCR, weight conversion ratio in kg feed/kg animal or L water/kg animal.

d European Medicines Agency (9).

e BW, body weight.

f Not applicable.

Specific decisions were required for doses for animals in specific age categories. Most antimicrobial products approved for use in feed and water did not differentiate doses by the age or production stage of the animal. However, some injectable and oral bolus product information included doses specific to young animals. For example, injectable gentamicin had doses for young pigs and chicks only (16). In addition, AMU data collected in Canada may be stratified by production stage in some species (e.g., farrowing, nursery or grow-finish stage in pigs). For this reason, we used these young animal doses to assign separate DDDvetCA specific to young animals, where applicable, which is a difference between our approach and that of EMA (9).

These young animal doses were often reported as a “per animal” dose. To obtain a dose in mg/kg these doses were divided by the average weight of the animal at treatment (16). For chicks and turkey poults, this weight was obtained from expert opinion (0.042 and 0.06 kg, respectively) (Agnes Agunos, personal communication, 2016). For piglets, we used ESVAC's standard piglet weight (4 kg) (9), which we confirmed to be consistent with Canadian pig production by expert opinion (Anne Deckert, personal communication, 2016). These young animal DDDvetCAs were labeled as such, to identify them as separate from the regular DDDvetCAs.

Another decision was required for handling products containing a mixture of various ingredients, such as anti-diarrheals, vitamins, minerals, and other additives, combined with very low doses of antimicrobials (16). The degree to which these products are used in pig production is not known, and with upcoming regulatory changes to require prescriptions for all medically important antimicrobials, these products may change or cease to be available (20). For these reasons, we assigned separate DDDvetCAs for the AAIs in these products. As with the young animal DDDvetCAs, the DDDvetCAs assigned using these low, or supplemental, doses were clearly labeled as such.

### Comparison Between DDDvetCAs and EMA's DDDvets

Decisions made by EMA and CIPARS differed in how the DDDs for animals were stratified by route of administration, making comparisons between the two sets of DDDs challenging (9). While acknowledging that the differences in stratification by route of administration could contribute to differences between the DDDvetCAs and the DDDvets, we compared feed, water and bolus DDDvetCAs to oral DDDvets that matched by AAI. We also compared injectable DDDvetCAs with parenteral DDDvets that matched by AAI.

Comparisons between DDDvetCAs and DDDvets were made by calculating the ratio of the DDDvetCA to the DDDvet using Equation (2) for each matching DDDvet by species and route of administration. Ratios of one (±10%) were considered equal. Ratios above 1.1 indicated the DDDvetCA was larger than DDDvet, while ratios below 0.9 indicated the DDDvetCA was smaller. Ratios above 1.5 and below 0.5, indicating a more than 50% disparity between the two values, were considered to indicate a substantial difference between the two standards.

(2) $$\begin{array}{r} Ratio\;DDDvetCA:DDDvet=\;DDDvetCA/DDDvet \end{array}$$

## Results

### Poultry

An examination of the distribution of daily AAI doses showed that, for poultry, doses often varied widely for a given AAI (Table 2). An example of the variation in doses was sulfamethazine for administration through water, with a daily dose range of 143.8–335.4 mg/kg (Table 2). Sixty-seven percent of the DDDvetCAs included products with a single dose for all indications. Seventy-eight percent of the median and mean daily doses were equal; notable exceptions included amprolium in feed (mean dose = 20.7, median = 16.3, mg/kg/day) (Table 2). When the mean and median dose differed, the median dose was smaller than the mean dose.

**Table 2:** The minimum, maximum, and median doses for all antimicrobial active ingredients for which Canadian defined daily doses for animals (DDDvetCAs) were assigned for poultry, by route of administration, and the number of products used to assign each DDDvetCA.

**Route of admin**	**Antimicrobial active ingredient^a^^,^^b^**	**Minimum dose**	**Maximum dose**	**Median dose**	**Ratio mean:median dose^c^**	**Number of products**
Feed	Amprolium	13.3	32.5	16.3	1.27	1
Feed	Avilamycin	2.9	2.9	2.9	1.00	1
Feed	Bacitracin	7.2	13.1	10.1	1.00	3
Feed	Bambermycin (GP)	0.3	0.3	0.3	1.15	1
Feed	Chlortetracycline	7.2	28.6	14.3	1.17	3
Feed	Clopidol	16.3	16.3	16.3	1.00	1
Feed	Decoquinate	3.9	3.9	3.9	1.00	1
Feed	Diclazuril	0.1	0.1	0.1	1.00	1
Feed	Erythromycin	28.6	28.6	28.6	1.00	1
Feed	Halofuginone	0.4	0.4	0.4	1.00	1
Feed	Lasalocid	13.0	13.7	13.3	1.00	2
Feed	Maduramicin ammonium	0.7	0.7	0.7	1.00	1
Feed	Monensin	13.0	13.0	13.0	1.00	3
Feed	Narasin	9.1	9.1	9.1	1.00	2
Feed	Narasin_nicarbazin	5.2	5.2	5.2	1.00	1
Feed	Nicarbazin	10.4	26.0	16.3	1.06	1
Feed	Nicarbazin_narasin	5.2	5.2	5.2	1.00	1
Feed	Oxytetracycline	7.2	28.6	14.3	1.17	8
Feed	Benzylpenicillin (GP)	0.3	0.3	0.3	1.05	2
Feed	Procaine benzylpenicillin	5.4	5.4	5.4	1.00	2
Feed	Robenidine	4.3	4.3	4.3	1.00	1
Feed	Salinomycin	7.8	7.8	7.8	1.00	5
Feed	Semduramicin	3.3	3.3	3.3	1.00	1
Feed	Sulfadiazine_trimethoprim (ELDU)	10.8	10.8	10.8	1.00	1
Feed	Trimethoprim_sulfonamide (ELDU)	2.2	2.2	2.2	1.00	1
Feed	Tylosin	26.0	26.0	26.0	1.00	4
Feed	Virginiamycin	2.9	2.9	2.9	1.00	4
Feed	Zoalene (Dinitolmide)	16.3	24.3	20.3	1.00	1
Water	Amoxicillin	12.0	12.0	12.0	1.00	2
Water	Amprolium	55.2	55.2	55.2	1.00	1
Water	Apramycin (ELDU)	23.0	23.0	23.0	1.00	1
Water	Enrofloxacin (ELDU)	5.8	5.8	5.8	1.00	1
Water	Erythromycin	13.3	26.6	19.9	1.00	1
Water	Lincomycin	3.7	3.7	3.7	1.00	2
Water	Lincomycin_spectinomycin	63.8	63.8	63.8	1.00	2
Water	Neomycin	8.1	55.8	20.4	1.07	8
Water	Oxytetracycline	5.6	40.9	18.6	1.01	11
Water	Benzylpenicillin	41.0	41.0	41.0	1.00	4
Water	Benzylpenicillin (supp)	3.8	3.8	3.8	1.00	3
Water	Pyrimethamine_sulfaquinoxaline	3.4	3.4	3.4	1.00	1
Water	Spectinomycin_lincomycin	127.7	127.7	127.7	1.00	2
Water	Streptomycin (supp)	19.6	19.6	19.6	1.00	3
Water	Sulfamethazine	143.8	335.4	230.0	1.03	4
Water	Sulfaquinoxaline	58.4	87.5	72.9	1.00	2
Water	Sulfaquinoxaline_pyrimethamine	11.2	11.2	11.2	1.00	1
Water	Tetracycline	11.1	40.9	20.4	1.05	13
Water	Tylosin	28.8	115.0	71.9	1.00	2
Injectable	Ceftiofur (ELDU) (YA)	2.6	2.6	2.6	1.00	1
Injectable	Gentamicin (YA)	4.8	16.8	10.8	1.00	1
Injectable	Lincomycin_spectinomycin (ELDU) (YA)	6.0	6.0	6.0	1.00	1
Injectable	Spectinomycin_lincomycin (ELDU) (YA)	12.0	12.0	12.0	1.00	1

a *ELDU, based on known extra-label drug use doses in circumstances where an antimicrobial is not licensed for use in this species, yet surveillance data has documented the use of this antimicrobial; GP, based on growth promotion doses as no treatment/prevention doses exist; YA, based on doses indicated for young animals; supp, based on doses from multiple ingredient products with low doses of antimicrobials*.

b *Antimicrobial active ingredients written as: Active ingredient 1_active ingredient 2 = DDDvetCA for active ingredient 1 when used in combination with active ingredient 2*.

c *Ratio mean:median dose = DDDvetCA/median dose*.

#### Antimicrobial Products and AAIs

**Figure 2:**
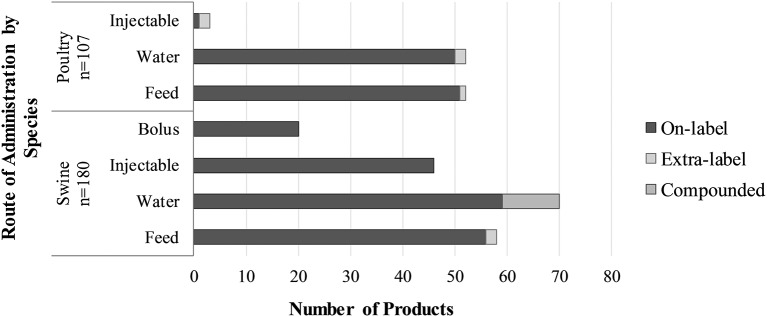
The number of antimicrobial products used to assign Canadian defined daily doses for animals (DDDvetCAs) for pigs and poultry, stratified by route of administration (feed, water, injectable, bolus).

**Figure 3:**
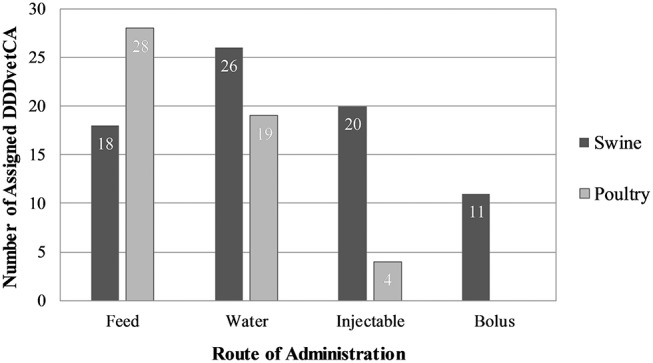
The number of assigned Canadian defined daily doses for animals (DDDvetCAs) by species and route of administration, including coccidiostats and ionophores.

The distribution of antimicrobial products by route of administration is illustrated in Figure 2. We did not identify any antimicrobial products for use by individual oral treatment (bolus), as poultry are generally not given individual oral treatments. Most products and AAI were for use in-feed (Figures 2, 3). Six in feed AAIs were ionophores and eight were synthetic coccidiostats. The AAI with the most products was oxytetracycline (19 products).

#### DDDvetCAs

The complete list of DDDvetCAs assigned for poultry can be found in Table 3. Feed was the route of administration with the most assigned DDDvetCAs (Figure 3). Two of the in feed DDDvetCAs (bambermycin and benzylpenicillin) were based only on growth promotion doses (Table 3). All four of the injectable DDDvetCAs were assigned for young chicks/poults only (Table 3). Three of these young animal injectable DDDvetCAs (ceftiofur, lincomycin-spectinomycin, spectinomycin-lincomycin) were based on extra-label drug use (ELDU) doses as the injectable products containing these AAIs do not include doses for poultry in the product information, however, surveillance data indicate use in the hatcheries. Other DDDvetCAs assigned from extra-label use doses included enrofloxacin and apramycin in water, and trimethoprim-sulfadiazine in feed (Table 3). The DDDvetCA for injectable gentamicin was assigned based on subcutaneous doses for chicks and poults from the product information, although gentamicin may be used off-label *in-ovo*. No DDDvetCAs were assigned for poultry based on compounded product doses at this time (Table 3).

**Table 3:** The Canadian defined daily doses for animals (DDDvetCA) in mg/kg_poultry_/day for antimicrobials used in poultry production, by antimicrobial active ingredient and route of administration.

**Antimicrobial active ingredient^a^^,^^b^**	**DDDvetCA (mg/kg/day)**
**FEED**	
Amprolium	20.7
Avilamycin	2.9
Bacitracin	10.1
Bambermycin (GP)	0.3
Chlortetracycline	16.7
Clopidol	16.3
Decoquinate	3.9
Diclazuril	0.1
Erythromycin	28.6
Halofuginone	0.4
Lasalocid	13.3
Maduramicin ammonium	0.7
Monensin	13
Narasin	9.1
Narasin_nicarbazin	5.2
Nicarbazin	17.2
Nicarbazin_narasin	5.2
Oxytetracycline	16.7
Penicillin G (GP)	0.3
Procaine penicillin G	5.4
Robenidine	4.3
Salinomycin	7.8
Semduramicin	3.3
Sulfadiazine_trimethoprim (ELDU)	10.8
Trimethoprim_sulfadiazine (ELDU)	2.2
Tylosin	26
Virginiamycin	2.9
Zoalene (Dinitolmide)	20.3
**WATER**	
Amoxicillin	12
Amprolium	55.2
Apramycin (ELDU)	23
Enrofloxacin (ELDU)	5.8
Erythromycin	19.9
Lincomycin	3.7
Lincomycin_spectinomycin	63.8
Neomycin	27.3
Oxytetracycline	18.8
Penicillin G	41
Penicillin G (supp)	3.8
Pyrimethamine_sulfaquinoxaline	3.4
Spectinomycin_lincomycin	127.7
Streptomycin (supp)	19.6
Sulfamethazine	236.4
Sulfaquinoxaline	72.9
Sulfaquinoxaline_pyrimethamine	11.2
Tetracycline	21.4
Tylosin	71.9
**INJECTABLE**	
Ceftiofur (ELDU) (YA)	2.6
Gentamicin (YA)	10.8
Lincomycin_spectinomycin (ELDU) (YA)	6
Spectinomycin_lincomycin (ELDU) (YA)	12

a *DDDvetCA, Canadian defined daily dose for animals; LA, long-acting; YA, young animal doses; supp, supplements; ELDU, extra-label drug use; GP, growth promotion dose*.

b *Antimicrobial active ingredients written as: Active ingredient 1_active ingredient 2 = DDDvetCA for active ingredient 1 when used in combination with active ingredient 2*.

#### Comparison Between DDDvetCAs and EMA's DDDvets

In poultry, comparisons between DDDvetCAs and DDDvets were only possible for the feed and water routes of administration, as the European Union/European Economic Area countries do not have any parenteral antimicrobials products approved for poultry. Nineteen DDDvetCAs could be compared to DDDvets (Table 4). For 34 feed and water DDDvetCAs there were no corresponding DDDvets for comparison.

**Table 4:** The ratio of the Canadian defined daily doses for animals (DDDvetCA) by water, feed and oral bolus routes of administration to the European Medicine Agency's defined daily dose for animals (DDDvet) by the oral route of administration in poultry.

**Antimicrobial active ingredient^a^^,^^b^**	**DDDvetCA**	**Canadian ROA**	**DDDvet^c^**	**EMA ROA^d^**	**Ratio^e^**
Amoxicillin	12	Water	16	Oral	0.8
Apramycin	23	Water	81	Oral	**0.3**
Chlortetracycline	16.7	Feed	30	Oral	0.6
Enrofloxacin	5.8	Water	10	Oral	0.6
Erythromycin	28.6	Feed	20	Oral	1.4
Erythromycin	19.9	Water	20	Oral	1.0
Lincomycin	3.7	Water	8.6	Oral	**0.4**
Lincomycin_spectinomycin	63.8	Water	22	Oral	**2.9**
Neomycin	27.3	Water	24	Oral	1.1
Oxytetracycline	16.7	Feed	39	Oral	**0.4**
Oxytetracycline	18.8	Water	39	Oral	0.5
Spectinomycin_lincomycin	127.7	Water	38	Oral	**3.4**
Sulfadiazine_trimethoprim	10.8	Feed	34	Oral	**0.3**
Sulfamethazine	236.4	Water	182	Oral	1.3
Sulfaquinoxaline	72.9	Water	60	Oral	1.2
Tetracycline	21.4	Water	71	Oral	**0.3**
Trimethoprim_sulfadiazine	2.2	Feed	6.4	Oral	**0.3**
Tylosin	26	Feed	81	Oral	**0.3**
Tylosin	71.9	Water	81	Oral	0.9

*Ratios above 1.5 and below 0.5, indicating substantial differences in these standardized doses, are in bold print*.

a *Antimicrobial active ingredients written as: Active ingredient 1_active ingredient 2 = DDDvetCA for active ingredient 1 when used in combination with active ingredient 2*.

b *ELDU, extra-label drug use*.

c *uropean Medicines Agency (12)*.

d *The EMA combined in feed, in water and oral bolus routes of administration into one oral DDDvet*.

e *Ratio, DDDvetCA/DDDvet*.

Results of the comparison showed some similarities and differences between the two sets of DDD for animals. Table 5 shows the frequency and proportion of DDDvetCA that were larger, smaller, or equivalent to their corresponding DDDvet according to the DDDvetCA/DDDvet ratio. More specifically, the DDDvetCAs and the DDDvets for erythromycin, neomycin and tylosin in water were similar with ratios between 0.9 and 1.1. Overall for poultry, five (26%) of the DDDvetCAs were larger and 11 (58%) of the DDDvetCAs were smaller than the corresponding DDDvets. DDDvetCAs that were notably different from the DDDvet included lincomycin combined with spectinomycin. Overall, nine DDDvetCAs (47%) differed by more than 50% from the equivalent DDDvets, with ratios <0.5 or >1.5.

**Table 5:** The frequency and proportion of Canadian defined daily doses for animals (DDDvetCA) that were larger, smaller, or equivalent to their corresponding defined daily dose for animals (DDDvet^a^), by species and route of administration.

**Species**	**Route of administration**	**DDDvetCA:DDDvet Ratio >1.1 *N* (%)**	**DDDvetCA:DDDvet Ratio <0.9 *N* (%)**	**DDDvetCA:DDDvet Ratio ≥ 0.9 and ≤1.1 *N* (%)**
Poultry	Feed	1 (17)	5 (83)	0 (0)
Poultry	Water	4 (31)	6 (46)	3 (23)
Pigs	Feed	0 (0)	11 (100)	0 (0)
Pigs	Water	5 (29)	7 (41)	5 (29)
Pigs	Injectable	5 (29)	6 (35)	6 (35)
Pigs	Bolus^b^	2 (33)	3 (50)	1 (17)
Poultry and pigs	All routes of administration	17 (24)	38 (54)	15 (21)

*DDDvetCAs were considered larger when the ratio of the DDDvetCA/DDDvet was larger than 1.1, smaller when the ratio was <0.9, and equivalent when the ratio was equal to or between 0.9 and 1.1*.

a *European Medicines Agency (12)*.

b *Bolus, administered as individual oral treatment*.

#### Other Observations

An examination of the Health Canada categorization of all DDDvetCAs in poultry revealed two Health Canada Category I AAIs, namely ceftiofur and enrofloxacin, which are used extra-label in poultry. Thirteen AAIs were Category II, with the remainder in Categories III and IV, or uncategorized (19). Uncategorized AAIs included avilamycin (an orthosomycin antimicrobial), tiamulin (a pleuromutilin antimicrobial), pyrimethamine (an anti-protozoal usually combined with sulfaquinoxaline), and the chemical coccidiostats.

### Pigs

As observed for poultry, an examination of the distribution of daily AAI doses showed that, for pigs, doses often varied widely for a given AAI. Like poultry, an example of a wide difference between the minimum and maximum daily dose of an AAI in pig was sulfamethazine in water, with minimum and maximum daily doses of 7 and 135 mg/kg, respectively (Table 6). Oxytetracycline, chlortetracycline, and bacitracin in feed had mean daily doses that varied from the median by a ratio of >1.5 (Table 5). Eighty-one percent of the median daily doses were identical to the median. Like poultry, the median dose was smaller than the mean dose in pigs, except for tiamulin in feed and neomycin (supplemental) bolus (Table 6).

**Table 6:** The minimum, maximum, and median doses for all antimicrobial active ingredients for which Canadian defined daily doses for animals (DDDvetCAs) were assigned for pigs, by route of administration, and the number of products used to assign each DDDvetCA.

**Route of admin^**a**^**	**Antimicrobial active ingredient^**b**^, ^**c**^**	**Minimum dose**	**Maximum dose**	**Median dose**	**Ratio mean:median dose^**d**^**	**Number of products**
Feed	Avilamycin	3.2	3.2	3.2	1.00	1
Feed	Bacitracin	1.6	11.0	2.8	1.61	4
Feed	Bambermycin (ELDU) (GP)	0.1	0.1	0.1	1.00	1
Feed	Chlortetracycline	2.2	26.2	6.6	1.58	12
Feed	Lincomycin	1.8	8.8	4.4	1.14	6
Feed	Lincomycin_spectinomycin	0.9	0.9	0.9	1.00	2
Feed	Narasin (GP)	0.6	0.6	0.6	1.00	2
Feed	Oxytetracycline	2.0	22.0	4.4	1.73	13
Feed	Benzylpenicillin	0.6	2.2	1.1	1.18	6
Feed	Procaine benzylpenicillin (ELDU)	13.2	13.2	13.2	1.00	1
Feed	Salinomycin (GP)	1.0	1.0	1.0	1.00	2
Feed	Spectinomycin_lincomycin	0.9	0.9	0.9	1.00	2
Feed	Sulfamethazine	4.4	4.4	4.4	1.00	5
Feed	Tiamulin	1.5	8.8	6.3	0.90	4
Feed	Tilmicosin	8.0	16.0	12.0	1.00	2
Feed	Tylosin	1.8	4.4	3.1	1.00	7
Feed	Tylvalosin	1.7	1.7	1.7	1.00	1
Feed	Virginiamycin	2.2	4.4	3.3	1.00	4
Water	Amoxicillin	16.0	16.0	16.0	1.00	2
Water	Ampicillin (C)	20.0	20.0	20.0	1.00	1
Water	Apramycin	10.0	10.0	10.0	1.00	1
Water	Gentamicin (C)	1.1	1.1	1.1	1.00	1
Water	Lincomycin	3.3	3.3	3.3	1.00	2
Water	Lincomycin_spectinomycin	2.2	2.2	2.2	1.00	2
Water	Neomycin	7.0	17.8	12.5	1.00	9
Water	Oxytetracycline	5.0	33.3	13.6	1.07	11
Water	Benzylpenicillin	17.8	17.8	17.8	1.00	3
Water	Benzylpenicillin (supp) (GP)	3.6	3.6	3.6	1.00	3
Water	Phenoxymethylpenicillin (C)	18.4	38.0	28.2	1.00	3
Water	Spectinomycin_lincomycin	4.5	4.5	4.5	1.00	2
Water	Streptomycin (supp) (GP)	18.4	18.6	18.5	1.00	3
Water	Sulfadiazine_trimethoprim (C)	20.0	44.4	30.0	1.08	5
Water	Sulfamerazine (supp)	2.5	4.1	3.3	1.00	4
Water	Sulfamethazine	7.0	135.0	79.2	1.00	10
Water	Sulfamethazine (supp)	6.3	6.3	6.3	1.00	3
Water	Sulfapyridine	33.3	33.3	33.3	1.00	1
Water	Sulfathiazole	37.8	75.0	39.3	1.18	6
Water	Sulfathiazole (supp)	5.0	15.6	10.3	1.00	4
Water	Tetracycline	2.0	17.8	8.3	1.04	12
Water	Tiamulin	4.9	4.9	4.9	1.00	2
Water	Tilmicosin (C)	10.0	10.0	10.0	1.00	1
Water	Trimethoprim_sulfadiazine (C)	5.5	8.9	7.0	1.01	5
Water	Tylosin	8.3	25.0	16.7	1.00	2
Water	Tylvalosin	5.0	5.0	5.0	1.00	1
Injectable	Ampicillin	6.0	6.0	6.0	1.00	1
Injectable	Benzathine benzylpenicillin combi (LA)	1.2	1.2	1.2	1.00	1
Injectable	Ceftiofur	3.0	3.0	3.0	1.00	6
Injectable	Ceftiofur (LA)	1.0	1.0	1.0	1.00	1
Injectable	Enrofloxacin	7.5	7.5	7.5	1.00	1
Injectable	Florifenicol	7.5	7.5	7.5	1.00	1
Injectable	Gentamicin (YA)	1.3	1.3	1.3	1.00	1
Injectable	Lincomycin	10.0	10.0	10.0	1.00	2
Injectable	Oxytetracycline	5.0	6.7	5.9	1.00	13
Injectable	Oxytetracycline (YA)	12.5	16.7	14.6	1.00	3
Injectable	Procaine benzylpenicillin	12.0	15.0	13.5	1.00	7
Injectable	Procaine benzylpenicillin (LA)	6.7	6.7	6.7	1.00	2
Injectable	Procaine benzylpenicillin_combi (LA)	1.5	1.5	1.5	1.00	1
Injectable	Sulfadoxine_trimethoprim	13.3	13.3	13.3	1.00	5
Injectable	Sulfadoxine_trimethoprim (YA)	25.0	25.0	25.0	1.00	5
Injectable	Tiamulin	11.0	11.0	11.0	1.00	1
Injectable	Trimethoprim_sulfadoxine	2.4	2.4	2.4	1.00	5
Injectable	Trimethoprim_sulfadoxine (YA)	5.0	5.0	5.0	1.00	6
Injectable	Tulathromycin (LA)	0.3	0.3	0.3	1.00	2
Injectable	Tylosin	5.5	5.5	5.5	1.00	1
Bolus	Neomycin (supp) (YA)	5	12.5	10	0.92	2
Bolus	Neomycin (YA)	8.9	33.3	17.8	1.11	6
Bolus	Oxytetracycline (YA)	5	55	18.9	1.39	9
Bolus	Spectinomycin (YA)	12.5	25.0	18.8	1.00	2
Bolus	Succinylsulfathiazole (supp) (YA)	24.0	48.0	36.0	1.00	1
Bolus	Sulfaguanidine (YA)	83.8	83.8	83.8	1.00	2
Bolus	Sulfamethazine (YA)	48.8	187.5	118.1	1.00	2
Bolus	Sulfanilamide (YA)	73.1	73.1	73.1	1.00	1
Bolus	Sulfathiazole (YA)	41.8	73.1	57.4	1.00	3
Bolus	Tetracycline (YA)	12.8	17.8	15.3	1.00	2
Bolus	Toltrazuril (YA)	20.0	20.0	20.0	1.00	1

a *Bolus, administered as individual oral treatment*.

b *ELDU, based on known extra-label drug use doses; GP, based on growth promotion doses as no treatment/prevention doses exist; C, based on compounded drug doses; LA, long acting; YA, based on doses indicated for young animals; supp, based on doses from multiple ingredient products with low doses of antimicrobials*.

c *Antimicrobial active ingredients written as: Active ingredient 1_active ingredient 2 = DDDvetCA for active ingredient 1 when used in combination with active ingredient 2. Exception: Benzathine Benzylpenicillin combi = Benzathine Benzylpenicillin in combination with any other antimicrobial active ingredient*.

d *Ratio mean dose:median dose = DDDvetCA/median dose*.

#### Antimicrobial Products and AAIs

The distribution of antimicrobial products by route of administration is illustrated in Figure 2. Most products (including ELDU and compounded products) and AAIs were for use in water (Figures 2, 3). Two in feed AAIs were ionophores and one was a synthetic coccidiostat. Like poultry, the AAI for which there were the most products was oxytetracycline (36 products).

#### DDDvetCAs

The complete list of assigned DDDvetCAs for pigs can be found in Table 7. The route of administration with the most assigned DDDvetCAs was in water (Figure 3). Two in water and three in feed DDDvetCAs were assigned based on growth promotion doses only, as these AAIs lacked doses for treatment or prevention (Table 7). Young animal DDDvetCAs were assigned for eleven bolus and four injectable AAIs (Table 7). DDDvetCAs were assigned from ELDU doses for bambermycin and procaine benzylpenicillin administered through feed, as the in-feed products containing these AAIs do not include doses for pigs in their product information, however, surveillance indicates use by this route of administration. Unlike poultry, some DDDvetCAs were assigned using compounded product doses (Table 7).

**Table 7:** The Canadian defined daily doses for animals (DDDvetCA) in mg/kg_pig_/day for antimicrobials used in pig production, by antimicrobial active ingredient and route of administration.

**Antimicrobial active ingredient^a^^,^^b^**	**DDDvetCA (mg/kg/day)**
**FEED**	
Avilamycin	3.2
Bacitracin	4.5
Bambermycin (ELDU) (GP)	0.1
Chlortetracycline	10.4
Lincomycin	5.0
Lincomycin_spectinomycin	0.9
Narasin (GP)	0.6
Oxytetracycline	7.6
Penicillin G	1.3
Procaine Penicillin G (ELDU)	13.2
Salinomycin (GP)	1.0
Spectinomycin_lincomycin	0.9
Sulfamethazine	4.4
Tiamulin	5.7
Tilmicosin	12.0
Tylosin	3.1
Tylvalosin	1.7
Virginiamycin	3.3
**INJECTABLE**	
Ampicillin	6.0
Benzathine penicillin G-combi^c^ (LA)	1.2
Ceftiofur	3.0
Ceftiofur (LA)	1.0
Enrofloxacin	7.5
Florifenicol	7.5
Gentamicin (YA)	1.3
Lincomycin	10.0
Oxytetracycline	5.9
Oxytetracycline (YA)	14.6
Procaine Penicillin G	13.5
Procaine Penicillin G (LA)	6.7
Procaine Penicillin G_combi^c^ (LA)	1.5
Sulfadoxine_trimethoprim	13.3
Sulfadoxine_trimethoprim (YA)	25.0
Tiamulin	11.0
Trimethoprim_sulfadoxine	2.4
Trimethoprim_sulfadoxine (YA)	5.0
Tulathromycin (LA)	0.3
Tylosin	5.5
**WATER**	
Amoxicillin	16.0
Ampicillin (C)	20.0
Apramycin	10.0
Gentamicin (C)	1.1
Lincomycin	3.3
Lincomycin_spectinomycin	2.2
Neomycin	12.5
Oxytetracycline	14.6
Penicillin G	17.8
Penicillin G (supp) (GP)	3.6
Penicillin V (C)	28.2
Spectinomycin_lincomycin	4.5
Streptomycin (supp) (GP)	18.5
Sulfadiazine_trimethoprim (C)	32.4
Sulfamerazine (supp)	3.3
Sulfamethazine	79.0
Sulfamethazine (supp)	6.3
Sulfapyridine	33.3
Sulfathiazole	46.2
Sulfathiazole (supp)	10.3
Tetracycline	8.6
Tiamulin	4.9
Tilmicosin (C)	10.0
Trimethoprim_sulfadiazine (C)	7.1
Tylosin	16.7
Tylvalosin	5.0
**BOLUS**^d^	
Neomycin (supp) (YA)	9.2
Neomycin (YA)	19.7
Oxytetracycline (YA)	26.2
Spectinomycin (YA)	18.8
Succinylsulfathiazole (supp) (YA)	36.0
Sulfaguanidine (YA)	83.8
Sulfamethazine (YA)	118.1
Sulfanilamide (YA)	73.1
Sulfathiazole (YA)	57.4
Tetracycline (YA)	15.3
Toltrazuril (YA)	20.0

a *DDDvetCA, Canadian defined daily dose for animals; LA, long-acting; YA, young animal; supp, supplements; ELDU, extra-label drug use; GP, growth promotion; C, compounded drug use*.

b *Antimicrobial active ingredients written as: Active ingredient 1_active ingredient 2 = DDDvetCA for active ingredient 1 when used in combination with active ingredient 2*.

c *Benzathine Penicillin G-combi and Procaine Penicillin G-combi (LA): when combined with any other antimicrobial active ingredient*.

d *Administered as individual oral treatments*.

#### Comparison Between DDDvetCAs and EMA's DDDvets

In pigs, comparisons between DDDvetCAs and DDDvets was possible for all routes of administration. Fifty-one DDDvetCAs could be compared to DDDvets (Table 8). The remaining 24 DDDvetCAs did not have any corresponding DDDvet.

**Table 8:** The ratio of the Canadian defined daily doses for animals (DDDvetCA) by route of administration to the European Medicine Agency's defined daily dose for animals (DDDvet) in pigs.

**Antimicrobial active ingredient^a^**	**DDDvetCA**	**Canadian ROA**	**DDDvet^b^**	**EMA ROA^c^**	**Ratio^d^**
Amoxicillin	16.0	Water	17.0	Oral	0.9
Ampicillin	20.0^e^	Water	30.0	Oral	0.7
Ampicillin	6.0	Injectable	12.0	Parenteral	0.5
Apramycin	10.0	Water	9.0	Oral	1.1
Benzathine Penicillin G_combi (LA)	1.2	Injectable	5.4	Parenteral	**0.2**
Ceftiofur (LA)	1.0	Injectable	0.8	Parenteral	1.3
Ceftiofur	3.0	Injectable	3.0	Parenteral	1.0
Chlortetracycline	10.4	Feed	31.0	Oral	**0.3**
Enrofloxacin	7.5	Injectable	3.4	Parenteral	**2.2**
Florifenicol	7.5	Injectable	9.5	Parenteral	0.8
Gentamicin	1.1^e^	Water	1.4	Oral	0.8
Gentamicin	1.3^f^	Injectable	1.4	Parenteral	0.9
Lincomycin	5.0	Feed	7.6	Oral	0.7
Lincomycin	10.0	Injectable	10.0	Parenteral	1.0
Lincomycin	3.3	Water	7.6	Oral	**0.4**
Lincomycin_spectinomycin	0.9	Feed	2.2	Oral	**0.4**
Lincomycin_spectinomycin	2.2	Water	2.2	Oral	1.0
Neomycin	19.7^f^	Bolus	25.0	Oral	0.8
Neomycin	12.5	Water	25.0	Oral	0.5
Oxytetracycline	26.2^f^	Bolus	26.0	Oral	1.0
Oxytetracycline	14.6^f^	Injectable	7.5	Parenteral	**1.9**
Oxytetracycline	7.6	Feed	26.0	Oral	**0.3**
Oxytetracycline	5.9	Injectable	7.5	Parenteral	0.8
Oxytetracycline	14.6	Water	26.0	Oral	0.6
Penicillin G	1.3	Feed	48.0	Oral	**<0.1**
Penicillin G	17.8	Water	48.0	Oral	**0.4**
Procaine Penicillin G	13.5	Injectable	13.0	Parenteral	1.0
Spectinomycin	18.8^f^	Bolus	33.0	Oral	0.6
Spectinomycin_lincomycin	0.9	Feed	3.4	Oral	**0.3**
Spectinomycin_lincomycin	4.5	Water	3.4	Oral	1.3
Sulfadiazine_trimethorprim	32.4^e^	Water	23.0	Oral	1.4
Sulfadoxine_trimethoprim	13.6^f^	Injectable	14.0	Oral	1.0
Sulfaguanidine	83.8^f^	Bolus	54.0	Oral	**1.6**
Sulfamethazine	118.1^f^	Bolus	92.0	Oral	1.3
Sulfamethazine	4.4	Feed	92.0	Oral	**<0.1**
Sulfamethazine	79.0	Water	92.0	Oral	0.9
Tetracycline	15.3^f^	Bolus	49.0	Oral	**0.3**
Tetracycline	8.6	Water	49.0	Oral	**0.2**
Tiamulin	5.7	Feed	9.7	Oral	0.6
Tiamulin	11.0	Injectable	12.0	Parenteral	0.9
Tiamulin	4.9	Water	9.7	Oral	0.5
Tilmicosin	10.0^e^	Water	15.0	Oral	0.7
Tilmicosin	12.0	Feed	15.0	Oral	0.8
Trimethoprim_sulfadiazine	7.1	Water	4.7	Oral	1.5
Trimethoprim_sulfadoxine	5.0^f^	Injectable	4.7	Parenteral	1.1
Trimethoprim_sulfadoxine	2.4	Injectable	3.0	Parenteral	0.8
Tylosin	3.1	Feed	12.0	Oral	**0.3**
Tylosin	5.5	Injectable	13.0	Parenteral	**0.4**
Tylosin	16.7	Water	12.0	Oral	1.4
Tylvalosin	1.7	Feed	3.6	Oral	0.5
Tylvalosin	5.0	Water	3.6	Oral	1.4

*Ratios above 1.5 and below 0.5, indicating substantial differences in these standardized doses, are in bold print*.

a *Antimicrobial active ingredients written as: Active ingredient 1_active ingredient 2 = DDDvetCA for active ingredient 1 when used in combination with active ingredient 2*.

b *European Medicines Agency. Defined daily doses for animals (DDDvet) and defined course doses for animals (DCDvet). 2016*.

c *The EMA combined in-feed, in-water and oral bolus routes of administration into one oral DDDvet*.

d *Ratio = DDDvetCA/DDDvet*.

e *DDDvetCA assigned using compounded doses*.

f *DDDvetCA assigned using young animal doses*.

As with poultry, results of the comparison showed some similarities and differences between the two sets of DDD for animals. Table 5 shows the frequency and proportion of DDDvetCA that were larger, smaller, or equivalent to their corresponding DDDvet using the DDDvetCA/DDDvet ratio. More specifically, DDDvetCAs and DDDvets were similar (±10%) for water administered amoxicillin, lincomycin-spectinomycin, apramycin, and sulfamethazine, and for injectable ceftiofur, lincomycin, procaine benzylpenicillin, sulfadoxine_trimethoprim, and tiamulin. All feed DDDvetCAs were smaller than their corresponding DDDvet (Table 5). Overall for pigs, a difference of more than 50% was observed between 35% of the DDDvetCAs and their corresponding DDDvets (e.g., enrofloxacin injectable DDDvetCA = 7.5 mg/kg/day; DDDvet = 3.4 mg/kg/day).

#### Other Observations in Pigs

An examination of the Health Canada categorization for all DDDvetCAs in pigs revealed that two Health Canada Category I AAIs, namely ceftiofur and enrofloxacin, were licensed for use in pigs (19). Sixteen AAIs were Category II, with the remainder in Categories III and IV, or uncategorized (as for poultry).

### Overall Results

Across both species, more DDDvetCAs were assigned for AAIs used in pigs than for poultry (Table 3). There were 53 feed, water and bolus DDDvetCAs that could be matched by AAI to 40 oral DDDvets, and 17 injectable DDDvetCAs that could be matched by AAI to 14 parenteral DDDvets (Tables 5, 8).

## Discussion

### Assigning DDDvetCAs

Assigning DDDvetCAs was a resource intensive and iterative process, and regular group discussions were needed to make a range of operational decisions. Examples of these decisions include, among others, the setup of the spreadsheet used to collect product information, how to interpret and handle various product information situations (such as combination products or dose ranges), and which doses to use for the determination of the mean dose (e.g., all doses or only unique doses). These decisions were sometimes revisited with the acquisition of new information.

Part of what made the DDDvetCA assignment resource intensive was the need for human resources to extract dose information from product information in the CVP and CMIB. At times, tracking down manufacturer and/or expert opinion was necessary where no licensed product dose information was available. Between 10 and 15 min were required to extract the required information from each product, provided the product information was comprehensive and clear. However, differences in the way product information and drug doses were written caused significant variation in the time needed to extract the information required. Some product information was clear and easy to understand, while others were more complex. Some products had doses for multiple indications, multiple species, or multiple routes of administration. For example, some water products included doses for individual animal dosing and for herd/flock dosing. The process became faster as familiarity with product information increased.

The decision to exclude growth promotion doses from the assignment of DDDvetCAs was consistent with EMA's guidelines, as in the European Union, the use of antimicrobial products for growth promotion is not permitted, and as a result, EMA's DDDvets are based on treatment and prevention doses only (9). In Canada, as of December 1, 2018, all antimicrobial products considered medically important by Health Canada will no longer be labeled for growth promotion purposes (20). By excluding growth promotion doses, the DDDvetCAs will remain relevant after this change. The decision to assign separate DDDvetCAs to growth promotion AAIs was based on the need to quantify their use, as these AAIs appear in Canadian surveillance data.

A departure from EMA's guidelines was the assignment of DDDvetCAs to AAIs used in an extra-label manner in the species of interest. Prescribing antimicrobials in an extra-label manner is legal for veterinarians in Canada, when no approved product for the species of interest exists (21). For the same reason that DDDvetCAs were needed for growth promotion AAIs, DDDvetCAs were needed for ELDU AAIs where surveillance data indicated their use in Canada. Since these extra-label DDDvetCAs are based on used doses, rather than labeled doses, they more closely resemble used daily doses (22). We recognize that assigning DDDvetCAs to these extra-label AAIs was a departure from defined daily dose methodology, however, due to the need to quantify the use of these AAIs we decided to include them in the DDDvetCA assignment.

We followed EMA's DDDvet guidelines for assigning separate DDDvetCAs to AAIs used in combination formulations, when their mean daily doses differed from single ingredient formulations (9). In contrast, the World Health Organization's methodology for the assignment of human DDDs assigns a single DDD to AAIs used in combination, using the mean daily dose of the main AAI ingredient only (23). When combination AAI products are used, the World Health Organization's method will only account for the use of the main ingredient, while CIPARS' (and EMA's) method will account for the use of each of the AAI in the combination product. This will ensure that all AAI use is considered for future modeling with AMR data.

The decision to use an average, or mean, daily dose to assign DDDvetCAs was also consistent with EMA's guidelines and with the DDD in human medicine (9, 10). While examining the distribution of AAI doses, we investigated using the median daily dose to assign DDDvetCAs. Over 80% of mean and median daily doses were identical in each species, so whether the mean or median dose was used made little difference to the resulting DDDvetCAs. Where differences existed, the mean was almost always larger than the median, which suggested that for these cases there may be some high dose outliers influencing the mean. Using the mean daily dose kept the DDDvetCAs more closely aligned with the EMA's methodology and the definition of a DDDvet (9). Examining AMU farm surveillance data to see if these outlying doses are in use may prove interesting.

### Differences Between DDDvetCAs and EMA's DDDvets

A major difference between DDDvetCAs and DDDvets is the stratification by routes of administration for products administered orally. EMA grouped the oral routes of administration together when assigning DDDvets, creating one category called oral (9, 12), while at CIPARS, we assigned DDDvetCAs to each oral route of administration separately. This difference in stratification very likely contributed to the differences between the feed, water and bolus DDDvetCAs and the oral DDDvets. Assigning DDDvetCAs separately to each oral route of administration will enable CIPARS to monitor changes in use between these routes of administration. An argument could be made that the DDDvetCAs should not be compared to DDDvets, due to the differences in route of administration stratification. However, we felt that these comparisons would be made by others, and by including the comparison in this study we could emphasize the strengths and limitations of doing so. The differences found between our feed, water and bolus DDDvetCAs and the oral DDDvets may have been less evident if we combined the oral routes of administration together in a similar manner to EMA. Even with the differences in stratification, some of the feed, water and bolus DDDvetCAs were identical or very close to the corresponding oral DDDvets.

CIPARS' method of assigning DDDvetCAs by using only unique AAI doses to calculate the mean daily dose also differed from EMA's guidelines (9). EMA's method of using the minimum and maximum daily doses to determine the mean daily dose meant that the doses on either end of the dose range had a greater effect on the mean. In contrast, by using the range of unique doses, any doses in the middle of the range have a moderating effect on the mean dose. For example, the unique daily doses for chlortetracycline in feed are 55, 110, 220, and 656 mg per kg of feed. If we used EMA's method, the mean daily dose would be 355.5 mg per kg of feed and a DDDvet of 14.2 mg/kg/day, while using our method results in a mean daily dose of 260.3 mg/kg feed and a DDDvetCA of 10.4 mg/kg/day.

An example of yet another way of calculating the mean daily dose is Postma et al.'s (13) method of averaging every dose found, regardless of how often each dose appears in product information. Postma et al. (13) felt this method was the clearest but acknowledged that the number of products that contained a specific AAI influenced the mean. Since the number of products containing an AAI does not necessarily reflect the frequency of use, we opted for a more neutral approach and attributed equal weight to every unique dose reported in the CVP and CMIB (16, 17).

Another difference between CIPARS', EMA's, and Postma et al.'s (13) methodology is in the approach to young animal doses. EMA included both young and adult doses in the calculation of a single average daily dose that could then be applied to all ages of animals (9). Postma et al. (13) followed human medicine methodology by incorporating only adult doses in the assignment of their DDDvets. CIPARS chose to separate young animal doses from the rest and assign age stratified DDDvetCAs in those AAIs with young animal doses. This decision was made because age-stratified AMU data were available to CIPARS, or would be available in the future, making age-stratified DDDvetCAs useful.

Defined daily dose methodology in human medicine deals with differences in dosing by age by incorporating the weight of a standard adult (70 kg) into the assignment of the DDD (10). As a result, human DDD are assigned in mg/day, rather than mg/kg/day as in veterinary medicine (10). Consequently, the World Health Organization's guidelines for ATC classification and DDD assignment in humans states that DDDs in children ages >1 month to 18 years are impossible to assign, as pediatric doses are dependent on age and weight, which vary widely (10). An advantage of assigning DDD for animals in mg/kg/day rather than mg/day is that they can be applied to animals in various weight and/or age categories. Assigning specific young animal DDDvetCAs, where young animal doses exist, can help us avoid the challenges experienced in human medicine when measuring AMU in pediatrics (24).

There are many other reasons for the observed differences in DDD for animals between CIPARS and EMA, one of which is that EMA may have had a wider range of AAI doses to work with, due to the collection of AAI doses from nine European countries (9). However, fully elucidating all the reasons for the differences between the EMA's DDDvets and the DDDvetCAs was outside the scope of this project. We can speculate that different labeling regulations, different treatment indications, and different husbandry practices may all contribute. Ultimately, whether the DDDvet or the DDDvetCA for an AAI is higher or lower does not necessarily reflect the use of that AAI in practice. DDD for animals are intended to be a technical measurement only (9). They are useful when standardized doses are needed for monitoring of trends in AMU and other purposes, in a variety of populations, whether they be national or regional.

### The Need for DDDvetCAs

The findings from this project confirmed the need for national DDDvetCAs for Canada for a few reasons. One reason was the observation that the DDDvets did not cover all the AAIs used in veterinary medicine in Canada. Also, while drawing conclusions from differences between DDDvetCAs and DDDvets assigned to oral routes of administration is difficult due to issues previously discussed, the differences observed between injectable DDDvetCAs and parenteral DDDvets appear to confirm the need for DDDvetCA that reflect antimicrobial selection pressure in a Canadian context.

The assigned DDDvetCAs have already been used by CIPARS for reporting farm-level surveillance data (5, 25). In the annual CIPARS report, the DDDvetCAs were used in the calculation of dose-based AMU indicators such as the number of defined daily doses for animals per 1,000 animal-days (26), and the number of defined daily doses for animals per population correction unit (5). Indicators such as these that use the DDDvetCAs will be valuable for in-country application to Canadian AMU data.

However, when comparing AMU between countries, using country specific DDD for animals such as the DDDvetCAs, may not be appropriate, due to the same challenges we observed when comparing DDDvetCAs and DDDvets. Differences in methodology and in antimicrobials authorized for use, among other issues, means that when reporting AMU internationally, it would be preferable for all reporting countries to use a set of international DDD for animals assigned using a single methodology. Ideally, these international DDD for animals would be assigned from globally represented product doses. Hence, the objectives of the reporting, whether national or international, will determine the choice of whether to use country-specific or international DDD for animals.

### Limitations

A limitation of the DDDvet methodology is that they are based on AAI doses from product information, which may not reflect the use of the AAI in practice (9, 10). When measuring AMU from surveillance data, where dosing practices may vary widely, the assigned DDDvets provide a consistent and transparent technical method for adjusting weight-based measures of AMU by dose. Where more specific information on AMU exposure is required, using used daily doses (UDD) may be more appropriate, noting that the results obtained from such an analysis will specific to the population from which the UDD were determined (22). Using UDD would require detailed data, including used doses and animal weights at treatment, and the results would be specific to a population at a point in time, as used doses and dosing practices frequently change.

The DDDvetCAs will need to be reviewed periodically as product doses may change, new products may be registered, or older ones discontinued. Also, new indications for use may be added to product information and changes in approved species may occur. While a DDDvetCA may be subject to review in specific instances, the aim is for the assigned DDDvetCAs to remain stable over time. This stability over time will allow for AMU trends to be followed long-term without frequent changes that will complicate analyses and interpretation. Future revisions will be aided by the Microsoft Excel® 2010 spreadsheet designed and used for tabulation and calculation of the DDDvetCAs, which will function as a database. To make future revisions easier, the development of an automated product registration system to flag product dose changes or new products would be helpful.

## Conclusion

The study of AMU is essential, enabling the examination of the impact on animal and human health due to the extent, nature, and determinants of AMU, and due to the associations between AMU and AMR. The DDDvetCAs will be valuable in the study of AMU in Canada, and while the process of assigning DDDvetCAs for the first time was challenging and resource intensive, maintaining them will require fewer resources. EMA's published principles for assigning DDDvets were an invaluable source of guidance and information for the creation of DDDvetCAs (9). Future steps for CIPARS include exploring DDDvetCA assignment for other production types such as cattle (beef and dairy), veal, and farmed fish.

## Data Availability

The datasets generated for this study are available on request to the corresponding author.

## Author Contributions

All authors listed have made a substantial, direct and intellectual contribution to the work, and approved it for publication.

### Conflict of Interest Statement

The authors declare that the research was conducted in the absence of any commercial or financial relationships that could be construed as a potential conflict of interest.

## Abbreviations

AMU: antimicrobial use
AMR: antimicrobial resistance
AAI: active antimicrobial ingredient
CIPARS: Canadian Integrated Program for Antimicrobial Resistance Surveillance
DDD: defined daily dose
DDD for animals: defined daily doses for animals
DDDvetCA: Canadian defined daily doses for animals
DDDvet: European Medicines Agency defined daily doses for animals
EMA: European Medicines Agency
ESVAC: European Surveillance of Veterinary Antimicrobial Consumption.

## References

[1] Rushton J Pinto Ferreira J Stärk KDC Antimicrobial resistance: the use of antimicrobials in the livestock sector. In: OECD Food, Agriculture and Fisheries Papers, Paris: OECD Publishing (2014). 10.1787/5jxvl3dwk3f0-en

[2] O'Neill J The Review on Antimicrobial Resistance. Tackling Drug-resistant Infections Globally: Final Report and Recommendations (2016).

[3] MARAN Nethmap NethMap 2017: Consumption of Antimicrobial Agents and Antimicrobial Resistance among Medically Important Bacteria in the Netherlands/MARAN 2017: Monitoring of Antimicrobial Resistance and Antibiotic Usage in Animals in the Netherlands in 2016 (2017).

[4] DANMAP DANMAP 2016—Use of Antimicrobial Agents and Occurrence of Antimicrobial Resistance in Bacteria from Food Animals, Food and Humans in Denmark (2017).

[5] Government of Canada Canadian Integrated Program for Antimicrobial Resistance Surveillance (CIPARS) 2016 Annual Report (2018).

[6] European Surveillance of Veterinary Antimicrobial Consumption ESVAC: Vision, Strategy and Objectives 2016–2020. London: European Medicines Agency (2017).

[7] Government of Canada Antimicrobial Resistance and Use in Canada: A Federal Framework for Action. Ottawa, ON: Public Health Agency of Canada (2014). 10.14745/ccdr.v40is2a01 PMC7009438 32077447

[8] World Health Organization. Global Action Plan on Antimicrobial Resistance. Geneva: World Health Organization (2015). 10.7196/samj.9644 26242647

[9] European Medicines Agency Principles on Assignment of Defined Daily Dose for Animals (DDDvet) and Defined Course Dose for Animals (DCDvet) (2015).

[10] WHO Collaborating Centre for Drug Statistics Methodology Guidelines for ATC Classification and DDD Assignment, 2019 (2018).

[11] AliAbadi FS Lees P. Antibiotic treatment for animals: effect on bacterial population and dosage regimen optimisation. Int J Antimicrob Agents. (2000) 14:307–13. 10.1016/S0924-8579(00)00142-4 10794952

[12] European Medicines Agency Defined Daily Doses for Animals (DDDvet) and Defined Course Doses for Animals (DCDvet) (2016).

[13] Postma M Sjölund M Collineau L Lösken S Stärk KDC Dewulf J. Assigning defined daily doses animal: A European multi-country experience for antimicrobial products authorized for usage in pigs. J Antimicrob Chemother. (2015) 70:294–302. 10.1093/jac/dku347 25223972

[14] MARAN 2018 Monitoring of Antimicrobial Resistance and Antibiotic Usage in Animals in the Netherlands in 2017 (2018).

[15] DANMAP 2017 Use of Antimicrobial Agents and Occurrence of Antimicrobial Resistance in Bacteria from Food Animals, Food and Humans in Denmark (2018).

[16] Canadian Animal Health Institute Compendium of Veterinary Products, Canadian ed. Hensall, ON: A.J. Bayley (2016).

[17] Canadian Food Inspection Agency Compendium of Medicating Ingredient Brochures. (2018). Available online at: http://www.inspection.gc.ca/animals/feeds/medicating-ingredients/eng/1300212600464/1320602461227 (accessed April 4, 2018).

[18] European Medicines Agency Sales of Veterinary Antimicrobial Agents in 30 European Countries in 2016—Trends from 2010 to 2016, Eighth ESVAC Report. (2018).

[19] Health Canada Categorization of Antimicrobial Drugs Based on Importance in Human Medicine. (2009). Available online at: https://www.canada.ca/en/health-canada/services/drugs-health-products/veterinary-drugs/antimicrobial-resistance/categorization-antimicrobial-drugs-based-importance-human-medicine.html (accessed July 13, 2016).

[20] Health Canada's Veterinary Drugs Directorate Notice to Stakeholders: Collaborative Efforts to Promote the Judicious Use of Medically-important Antimicrobial Drugs in Food Animal Production. (2014). Available online at: https://www.canada.ca/en/health-canada/services/drugs-health-products/veterinary-drugs/antimicrobial-resistance/notice-stakeholders-collaborative-efforts-promote-judicious-use-medically-important-antimicrobial-drugs-food-animal-production.html (accessed August 11, 2017).

[21] Health Canada's Veterinary Drugs Directorate Policy on Extra-Label Drug Use (ELDU) in Food Producing Animals. (2008). Available online at: https://www.canada.ca/en/health-canada/services/drugs-health-products/veterinary-drugs/extra-label-drug-use/policy-extra-label-drug-use-eldu-food-producing-animals.html (accessed January 30, 2019).

[22] Timmerman T Dewulf J Catry B Feyen B Opsomer G de Kruif A. Quantification and evaluation of antimicrobial drug use in group treatments for fattening pigs in Belgium. Prev Vet Med. (2006) 74:251–63. 10.1016/j.prevetmed.2005.10.003 16675051

[23] World Health Organization Definition and General Considerations of DDD. (2016). Available online at: https://www.whocc.no/ddd/definition_and_general_considera/ (accessed February 12, 2017).

[24] Gravatt LA Pakyz AL. Challenges in measuring antibiotic consumption. Curr Infect Dis Rep. (2013) 15:559–63. 10.1007/s11908-013-0374-9 24097249

[25] Agunos A Léger DF Carson CA Gow SP Bosman A Irwin RJ. Antimicrobial use surveillance in broiler chicken flocks in Canada, 2013–2015. PLoS ONE. (2017) 12:e0179384. 10.1371/journal.pone.0179384 28658278 PMC5489168

[26] Government of Canada Canadian Integrated Program for Antimicrobial Resistance Surveillance (CIPARS) 2015 Annual Report. (2016).

